# Sleep Related Epilepsy and Pharmacotherapy: An Insight

**DOI:** 10.3389/fphar.2018.01088

**Published:** 2018-09-27

**Authors:** Jaya Kumar, Amro Solaiman, Pasuk Mahakkanukrauh, Rashidi Mohamed, Srijit Das

**Affiliations:** ^1^Department of Physiology, Faculty of Medicine, Universiti Kebangsaan Malaysia Medical Centre, Kuala Lumpur, Malaysia; ^2^Department of Anatomy, Universiti Kebangsaan Malaysia Medical Centre, Kuala Lumpur, Malaysia; ^3^Department of Anatomy, Faculty of Medicine, Chiang Mai University, Chiang Mai, Thailand; ^4^Excellence Centre in Forensic Osteology Research Center, Faculty of Medicine, Chiang Mai University, Chiang Mai, Thailand; ^5^Department of Familty Medicine, Universiti Kebangsaan Malaysia Medical Centre, Kuala Lumpur, Malaysia

**Keywords:** epilepsy, sleep, seizure, SHE, BECTS, PS, rolandic, panayiotopoulos

## Abstract

In the last several decades, sleep-related epilepsy has drawn considerable attention among epileptologists and neuroscientists in the interest of new paradigms of the disease etiology, pathogenesis and management. Sleep-related epilepsy is nocturnal seizures that manifest solely during the sleep state. Sleep comprises two distinct stages i.e., non-rapid eye movement (NREM) and rapid eye movement (REM) that alternate every 90 min with NREM preceding REM. Current findings indicate that the sleep-related epilepsy manifests predominantly during the synchronized stages of sleep; NREM over REM stage. Sleep related hypermotor epilepsy (SHE), benign partial epilepsy with centrotemporal spikes or benign rolandic epilepsy (BECTS), and Panayiotopoulos Syndrome (PS) are three of the most frequently implicated epilepsies occurring during the sleep state. Although some familial types are described, others are seemingly sporadic occurrences. In the present review, we aim to discuss the predominance of sleep-related epilepsy during NREM, established familial links to the pathogenesis of SHE, BECTS and PS, and highlight the present available pharmacotherapy options.

## Introduction

Epilepsy is characterized by frequent and unpredictable disruptions of brain functions resulting in “epileptic seizures.” Epilepsy has a great impact on the quality of life through increased incidence of injury and death, unemployment rates, lower monthly incomes, higher household costs and high absenteeism at work and schools (Jennum et al., 2017; Trinka et al., 2018; Wibecan et al., 2018). An epileptic seizure is considered as a transient episode of signs or symptoms, including transitory confusion, staring speech, irrepressible jerking movements, loss of consciousness, psychic symptoms such as fear and anxiety, due to the abnormal synchronous neuronal activity of the brain. The International League Against Epilepsy (ILAE) published a recent clinical definition of epilepsy in which a patient with any of the following conditions is considered to be an epileptic i.e., (i) two or more unprovoked seizures within more than 24 h apart; (ii) one unprovoked seizure and a probability of further seizures similar to the general recurrence risk, occurring over the next 10 years; (iii) definite diagnosis of an epilepsy syndrome (Fisher et al., 2014). Genesis of epilepsy is attributed to various predispositions that include neurological, perceptive, psychological, and social factors, which could either stimulate or worsen the syndrome. In early 2017, the point prevalence of active epilepsy was found to be 6.38/1,000 individuals, while the lifetime prevalence was 7.60/1,000 persons. Meanwhile, the annual cumulative incidence of epilepsy was 67.77/100,000 persons and the incidence rate was 61.44/100,000 person-years. The active annual prevalence, prevalence during lifetime and the incidence of epilepsy were found to be higher in the developing countries (Fiest et al., 2017).

A systematic review revealed that epilepsies of unknown etiology had the highest prevalence compared to the epilepsies of known origin (Fiest et al., 2017). These were due to known underlying factors that cause seizures such as brain damage (Sizemore et al., 2018), metabolic diseases (Tumiene et al., 2018), infections (Bartolini et al., 2018), hemorrhagic stroke (Zhao et al., 2018), and gene mutations (Leonardi et al., 2018). These precipitating factors tilt the balance between excitatory and inhibitory neurotransmissions which has been established in different types of epilepsy. Physical and psychological comorbidities are usually accompanied with epilepsy, such as depression (Jamal-Omidi et al., 2018), sleep disorders (Castro et al., 2018), and body injuries (Mahler et al., 2018). Advanced cases may suffer from memory loss (Reyes et al., 2018), behavioral disorders (Jalihal et al., 2018), and disturbance of autonomic functions (Fialho et al., 2018). The rate of sudden death in epileptic patients was reported to be three times higher than non-epileptic individuals (Kothare and Trevathan, 2018; Pati et al., 2018).

Sleep deprivation is very common among the epileptic patients and lack of sleep could worsen the seizure expressions (Neto et al., 2016). In animal models, sleep deprivation was shown to heighten the propensity to seizures (McDermott et al., 2003). Sleep deprivation has been correlated with decline in various aspects of brain functional connectivity (Nilsonne et al., 2017). Generally, sleep deprivation is secondary to other factors such as illness, emotional or psychological stress, and alcohol use. Hence, lack of sleep alone may not be sufficient to cause seizures (Razavi and Fisher, 2017). A large body of literature on the effects of epilepsy on sleep and/or sleep-deprivation on the epileptic state has been collated (St Louis, 2011; Unterberger et al., 2015).

Sleep-related epilepsy represents nocturnal seizures that manifest solely during the sleep state (Tchopev et al., 2018). Approximately 12% epileptic patients are affected by sleep-related epilepsy with the majority suffering from focal epilepsy (Derry and Duncan, 2013; Losurdo et al., 2014). In a recent case report, focal epilepsies were anatomically linked to epileptogenic origins at the right frontal lobe, using white matter tractography MRI (Tchopev et al., 2018). In a separate study, ambulatory electroencephalogram (EEG) measurement in outpatient setting reported frontal lobe seizures to manifest more readily between 12 a.m. and 12 p.m., particularly around 6:30 a.m., whereas temporal lobe seizures expressed more frequently between 12 p.m. and 12 a.m., specifically around 8:50 p.m. (Pavlova et al., 2012). In addition to seizure onset, few seizures seem to propagate more readily during sleep, based on anatomical locus. Medial temporal lobe regions were shown more likely to manifest spike production or propagation during NREM sleep stage compared to other brain regions (Lambert et al., 2018). Sleep-related epilepsy is often misdiagnosed as sleep disorders (Tinuper and Bisulli, 2017), especially in cases where the seizures manifest exclusively during sleep. Over the past decade, the discovery of numerous pre-disposing genes and availability of advanced diagnostic tools have shed more light in understanding the nature of sleep-related epilepsy.

In the present review, we discuss sleep-related epilepsy with particular emphasis on three of the most frequently implicated epilepsies during the sleep state which include sleep related hypermotor epilepsy (SHE), benign partial epilepsy with centrotemporal spikes (BECTS), and Panayiotopoulos Syndrome (PS).

## Expression of seizures in NREM vs. REM sleep stages

In comparison with rapid eye movement (REM) sleep, the expression of focal seizure was 87 times more common in N1, 68 times more likely in N2, and 51 times more likely in N3. For generalized seizures, the seizure rate was 3.1 times higher in N1, 3.13 times higher in N2 and 6.59 times higher in N3 compared to the REM stage. Sleep-related epilepsies such as Benign Epilepsy of Childhood with rolandic spikes were common during the non-rapid eye movement (NREM) stages, especially during N3 (Ng and Pavlova, 2013), SHE was expressed more readily during N1/N2 (Nobili et al., 2014; Yeh and Schenck, 2014), and PS during N1 (Demirbilek and Dervent, 2004). Taken together, the existing literature suggests that sleep-related seizures are more likely to occur during the NREM stages of sleep.

NREM sleep is known as the state of neuronal synchronization, whereas REM as the most desynchronized sleep state. EEG findings suggest synchronization changes are more likely to take place during the transitions between the sleep states, rather than during the particular sleep states (Baghbani et al., 2018). In general, two types of synchronization exist; long-range (involves numerous brain regions) and local synchronization (involves adjacent neurons). During seizures, the long-range synchronization is impaired and local synchronization is enhanced as a result of the altered extracellular content of calcium (Ca^2+^) and potassium (K^+^) ions. In the pathogenesis of paroxysmal discharge, various predisposing factors (familial vs. sporadic) could alter the electrophysiological properties of numerous receptors, which may potentially decrease the extracellular level of Ca^2+^ and increase the extracellular content of K^+^, simultaneously (Amzica et al., 2002). Such changes inhibit synaptic transmission and propagation of action potential (Seigneur and Timofeev, 2011), which subsequently impair long-range synchronization and promote electrical coupling between cortical interneurons (Galarreta and Hestrin, 2001) and glial cells (Giaume and McCarthy, 1996). Long-range synchronization is also impaired during the slow-wave sleep (N3) (Ng and Pavlova, 2013). A preponderance of cortical slow oscillations takes place at this stage that results in a significant drop in the extracellular content of Ca^2+^, leading to high rates of synaptic failures (Steriade et al., 1993; Crochet et al., 2005). For instance, during the N3 stage, the mesenphalic reticular formation cholinergic neurons that allow transmission of impulses from the thalamus to the cortex are least active (less active during NREM stages) (Ng and Pavlova, 2013). As hypothesized by Timofeev et al. (2012) the significant drop in extracellular levels of Ca^2+^ during slow-wave sleep can promote the opening of hemichannels (Thimm et al., 2005) and electrical coupling between neighboring neurons (local synchrony) (Timofeev et al., 2012). Taken together, neuronal synchronization (local) along with pre-existing pro-epileptic conditions (such as channellopathies) seem to reinforce the predominance of seizure expressions during sleep state.

The seizure expressions during NREM stages are simplified in Table 1.

**Table 1:** Table showing the expression of seizures during NREM sleep.

**NREM sleep** **stage**	**Focal** **seizure^a^**	**Generalized** **seizure^a^**	**Sleep related** **epilepsy^a^**
N1	87	3.1	SHE, PS
N2	68	3.13	SHE
N3	51	6.59	BECTS

a *number of times focal seizure taking place during NREM compared to REM stage*.

*^a^number of times generalized seizure taking place during NREM compared to REM stage*.

*^a^predominance of specific sleep related epilepsy during the NREM stages*.

## Sleep related hypermotor epilepsy

SHE, or previously known as the Nocturnal Frontal Lobe Epilepsy (NFLE) is a type of sleep-related epilepsy with frontal and extrafrontal regions as seizure onset zones that characterized by peculiar motor aspects of seizures (Tinuper et al., 2016; Vignatelli et al., 2017). The hallmark feature of this rare partial epilepsy is motor seizures that manifest almost exclusively during the NREM stage of sleep. The familial form of this epilepsy, also widely known as the autosomal dominant NFLE manifests between the age 8 and 12 years (Picard and Scheffer, 2012). SHE is the first of its kind to be associated with a causative agent, which are mutations of 3 subunit genes of nicotinic acetylcholine receptors (nAChRs) (Steinlein et al., 1995; Marini and Guerrini, 2007); reported in approximately 10% of the affected families (Heron et al., 2007).

**Figure 1:**
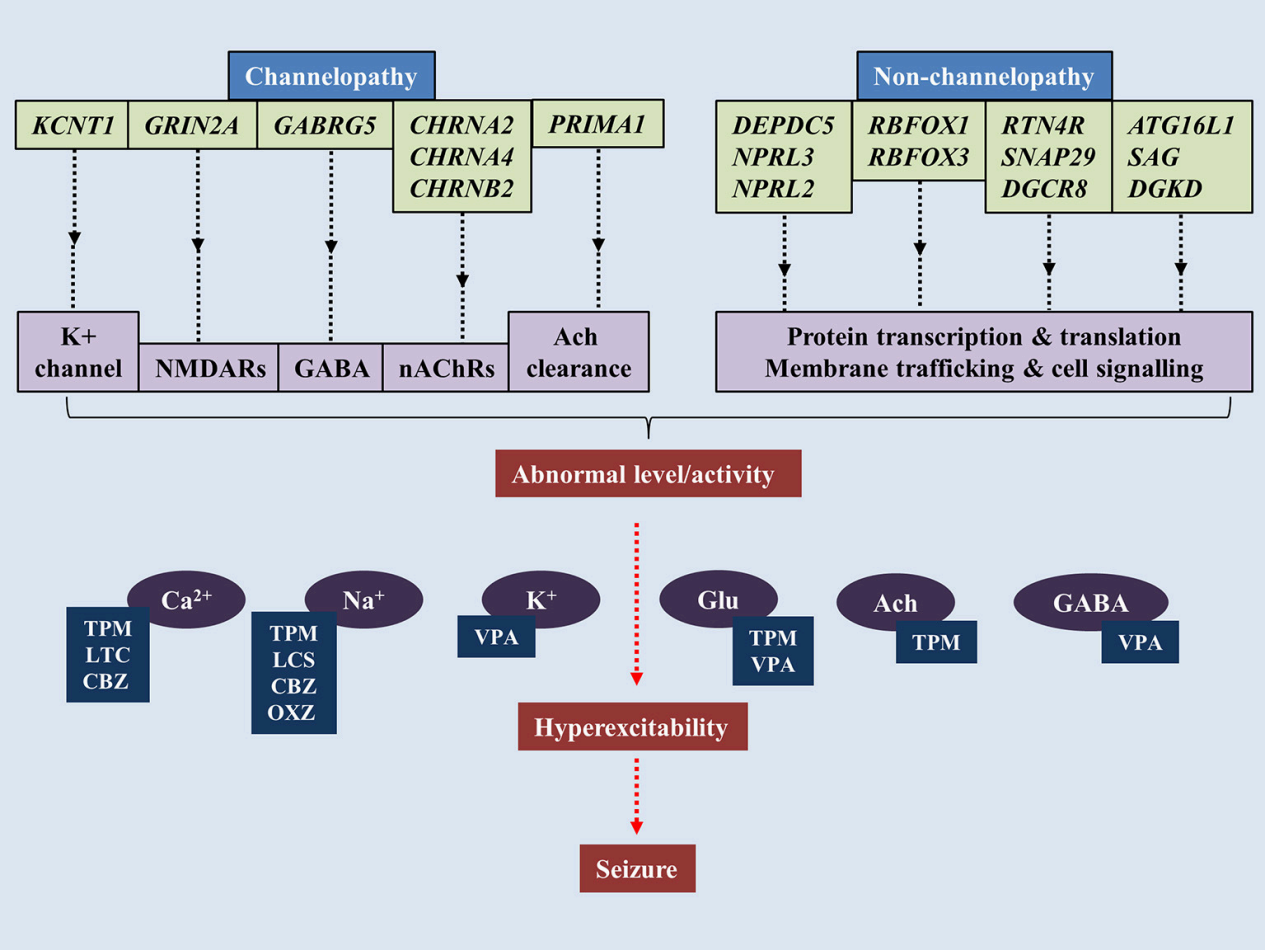
Pathogenesis of sleep-related epilepsy. Mutations of genes associated with channelopathy and non-channelopathy origin of sleep related epilepsy could disrupt the balance between inhibitory and excitatory neurotransmissions in central nervous system, leading to manifestation of seizure. Various anti-epileptic drugs alleviate seizure by restoring chemical balance in brain. TPM, topiramate; VPA, valproic acid; CBZ, carbamazepine; OXC, oxcarbazepine; LTC, levetiracetam; LCS, lacosamide.

The notion that SHE is a channelopathy is derived from early findings on mutations of nAChRs genes such as cholinergic receptor nicotinic alpha 2 subunit (*CHRNA2)*, cholinergic receptor nicotinic alpha 4 subunit (*CHRNA4)* and cholinergic receptor nicotinic beta 2 subunit *(CHRNB2)* encoding for nAChRs containing subunits of α4β2 or heteromers with subunits of α2/β2/β4 (Figure 1) (Di Resta et al., 2010; Wallace and Bertrand, 2013; Becchetti et al., 2015). The anatomical projections of cholinergic neurons from pons and basal forebrain toward thalamus and cortex have been implicated in the regulation of sleep-wake cycle (Saper et al., 2010). Existing literature suggests increased acetylcholine release during wakefulness and REM sleep, whereas marked decrease during NREM sleep (Jones, 2008). Findings from mutant murine models of SHE (expressing B2-V287L) showed altered sleep pattern and development of spontaneous seizures during the slow wave sleep state (O'Neill et al., 2013). Molecular results show that mutated nAChRs could be hyperfunctional, and thus maintaining abnormal gamma-Aminobutyric acid-(GABA)ergic and glutamatergic neurotransmission, even with very minimal acetylcholine (Ach) available to bind to (Aracri et al., 2010). Hyperfunction of nAChRs also could be due to longer duration of Ach remaining in the synapse. In a recent study, a novel autosomal recessive phenotype of SHE was identified in a two-generation Australian family of Italian origins. Whole genome sequencing revealed mutations of proline rich membrane anchor 1 (*PRIMA1)* on chromosome 14 that encodes for PRIMA1 transmembrane protein, which anchors acetylcholinesterase at synapses for hydrolysis of acetylcholine. The authors went on to point out that perturbations in the cholinergic responses attributed to dysfunctional acetylcholinesterase could alter central and peripheral process of seizure expressions and likely to transform to SHE (Hildebrand et al., 2015). More recently, the occurrence of sleep-related epilepsy in SHE has been directly related to dysregulation of GABAergic neurotransmission. The reversal potential of GABA_A_-mediated inhibitory post-synaptic potential requires the movement of chloride ions (Cl^−^) in and out of the cells, which are mediated by co-transporters such as Na^+^/K^+^/Cl^−^ co-transporter-1 (NKCC1) and K^+^/Cl^−^ co-transporter-2 (KCC2) (Kaila et al., 2014). In a murine model of SHE (expressing B2-V287L), delayed surface expression of KCC2 was reported in layer V of prefrontal cortex (PFC), which is the most susceptible part of PFC to epileptiform activities. The delay was noticed during the first postnatal weeks, which led the authors to suggest that PFC is more prone to neuronal network-related pathologies such as epilepsy (Amadeo et al., 2018). Neural network in layer V of PFC is predominantly regulated by nAChRs expressing β2 subunit (Poorthuis et al., 2012). Despite mutation at B2-V287L, the cell surface expression of nAChRs remained the same (Manfredi et al., 2009). Thus, functional changes were thought to be attributable to expression of B2-V287L (Amadeo et al., 2018). Altered Ca^2+^ signals following hyperactivity of nAChRs could upregulate the surface expression of KCC2 as a compensatory mechanism to counterbalance the overactive neuronal network by increasing the Cl^−^ turnover. Collectively, these lead to retardation of GABAergic switch in PFC and ultimately hyperexcitability (Amadeo et al., 2018).

In addition to nAChRs, mutations in *KCNT1* (gene encoding for potassium-sodium activated channel subfamily T member 1) were also linked to a rather severe form of SHE and sporadic SHE. The patients found with these mutations are also presented with various psychiatric features and intellectual disabilities, which was dissimilar to the usual form of SHE. The onset age for *KCNT1* mutation-related SHE was below the onset age of classical SHE and the penetrance of KCNT1 mutations are 100% (Heron et al., 2012) which is higher than classical SHE. *KCNT1* encodes for KCNT1 channel subunit which binds with potassium-sodium activated subfamily T member 2 (KCNT2) to form the heterotetrameric complex of the channel. The *KCNT1* mutations mostly affect the nicotinamide adenine dinucleotide interaction with C-terminal of the channel, which could disrupt the modulation of the channels' functions (Tamsett et al., 2009).

More recent findings have focused on the non-channelopathy-based pathogenesis of the SHE (Dibbens et al., 2013; Picard et al., 2014; Korenke et al., 2016). *DEPDC5* (Disheveled, Eg-10 and Pleckstrin Domain containing proteins) is a gene that encodes a protein structurally-related to Gap Activity Toward Rags 1 (GATOR1), which is an important negative modulator of mammalian target of Rapamycin (mTOR) Complex 1 (mTORC1) that regulates various cell functions (Bar-Peled et al., 2013). *DEPDC5* mutations have been implicated in familial temporal lobe epilepsy, SHE and familial focal epilepsy with variable loci (Dibbens et al., 2013; Ishida et al., 2013; Martin et al., 2014). Other genes expressing GATOR1 such as nitrogen permease regulator-like 2 (*NPRL2)* and nitrogen permease regulator-like 3 (*NPRL3)* were also linked to sporadic and familial form of epilepsy (Ricos et al., 2016). It was thought that mal-interaction between mTORC1 and GATOR1 may alter cortical neuroarchitecture as epileptic patients with *NPRL3* mutations were presented with dysplastic brain lesions (Sim et al., 2016). Nevertheless, few patients despite experiencing seizures showed no anomalies in brain imaging, suggesting the structural changes could be microscopic or other unknown pathway could mediate the pathogenesis (Korenke et al., 2016). From a functional perspective, reduced negative modulation of mTORC1 leads to overactivity of the protein complex, which has been demonstrated in epileptic brains (Sha et al., 2012; Sosunov et al., 2012). In addition, inhibition of mTORC1 has been shown to block epileptogenesis (Huang et al., 2010). Thus, it was hypothesized that hyperactivated mTORC1 signaling leads to rhythmic increase in neuronal excitability (Cho, 2012).

## Benign childhood epilepsy with centrotemporal spikes

BECTS, also known as Rolandic Epilepsy (RE) is the most common type of epilepsy syndrome in children. The typical onset age of BECTS is between 3 and 13 years, with spontaneous remission of seizures upon puberty (Berg et al., 2010). The hallmark feature of BECTS' EEG is high voltage spike and wave, mainly centrotemporal spikes. The seizures predominantly occur during NREM sleep and last for 1–3 min (Stephani, 2000).

Although initially described as idiopathic (Panayiotopoulos et al., 2008), several studies postulated a hereditary link to the disease (Vears et al., 2012; Shi et al., 2018). Numerous potential genes predisposing to BECTS were investigated to no avail (Neubauer et al., 1998; Strug et al., 2009; Pal et al., 2010). Proline-rich transmembrane protein gene (PRRT2) that was associated with paroxysmal kinesigenic dyskinesias (Chen W. J. et al., 2011) and RE (Dimassi et al., 2014), were screened in 9 cohorts of 53 sporadic patients and 250 controls in a mainland Chinese population. Genomic sequencing revealed no association between PRRT2 mutations and BECTS (Che et al., 2017). In a separate study, genes associated with epilepsy-aphasia spectrum, that encode for elongator acetyltransferase complex subunit 4 (ELP4) and sushi-repeat containing protein X-linked 2 (SRPX2) (Roll et al., 2006; Strug et al., 2009), were studied for their potential interactions in BECTS. The investigators utilized whole genome sequencing on 290 patients with European ancestry diagnosed with RE or atypical RE (ARE) in Germany, Canada and Austria and no pathological link between RE/ARE and ELP4 and SRPX2 genes were found (Reinthaler et al., 2014). The role of ELP4 in pathogenesis of BECTS was also downplayed by another study conducted in a Greek population (Gkampeta et al., 2014). The genetic risk factor in BECTS was identified in the gene that encodes for α2 subunit of N-methyl-D-aspartate receptors (NMDARs) (*GRIN2A)*. A mutational analysis carried out in 2 independent cohorts of 359 patients identified heterozygous mutations in 27 of 359 subjects and described exon-disrupting microdeletions in 3 of 286 individuals (Lemke et al., 2013). More recently, an unbiased gene-burden analysis of 194 patients against 567 in-house and 33370 online ExAC controls showed only GRIN2A rare CSDD15, CADD15 + LOF, and LOF variants were more frequent in BECTS (Bobbili et al., 2018). NMDARs are glutamate-bound excitatory receptors with important roles in synaptic transmission and plasticity (Paoletti, 2011). Numerous animal models have implicated altered NMDARs functions in development of epilepsy (Frasca et al., 2011; Di Maio et al., 2012). Thus, mutations in *GRIN2A* are thought to affect the electrophysiological property of GluN2A subunit containing NMDARs (Lemke et al., 2013). In addition to *GRIN2A*, genes *DEPDC5* (Lal et al., 2014), gamma-aminobutyric acid receptor subunit gamma-2 (*GABRG2)* (Reinthaler et al., 2015), RNA Binding Fox-1 Homolog 1 (*RBFOX1)*, RNA Binding Fox-1 Homolog 3 (*RBFOX3)* (Lal et al., 2013) and *KCNT1* (Shi et al., 2018) were also implicated in BECTS.

Persistent neuropsychiatric deficits in executive functions, intelligence and attention have been reported in BECTS patients despite spontaneous remission during the adolescence (Currie et al., 2018; Ofer et al., 2018). Myriad of neuroimaging studies associated the cognitive decline with abnormal cortical changes (Overvliet et al., 2013; Pardoe et al., 2013). Generally, cortical gray matter decreases over time from childhood to young adulthood (Shaw et al., 2006). However, in the BECTS population, the changes in cortical thickness were greater compared to the control group and there was also delay in reaching the normative values. This may explain the persistence of language problems in BECTS patients even after the remission (Pardoe et al., 2013). On the contrary, a more recent study precluded any direct relationship between centrotemporal spikes frequency and morphological changes in cortex of BECTS patients. However, one particular region, R pars opercularis showed thinner cortex in BECTS children relating the atypical cortical features with poor processing speed (Fujiwara et al., 2018). Several studies have shown BECTS to predominantly affect the left hemisphere of the brain, linking BECTS to language dysfunction seen in the children (Overvliet et al., 2013). This was further corroborated by the discovery of widespread white matter abnormalities confining to the left hemisphere of BECTS children with intellectual disabilities, particularly verbal IQ (Kim et al., 2014). In addition to intellectual disabilities, left hemisphere anomaly was also correlated with greater social fear among BECTS patients (Potegal et al., 2018). More recently, the network re-organization leading to cognitive dysfunctions in BECTS was thought to originate from right homologous brain areas. In treatment naïve, early-onset BECTS patients, escalated brain activity was seen in the right Broca's area during the early stage of the disease, suggesting the compensatory change to take place in the right hemisphere of the brain (Chen et al., 2018). Subcortical structures such as the basal ganglia, sensorimotor networks, and striato-cortical circuitry were also shown to have epilepsy-related functional connectivity with BECTS (Li et al., 2017).

## Panayiotopoulos syndrome

PS is a common idiopathic childhood epilepsy syndrome with predominant features of autonomic signs. The majority of the children were found to be in the age group of 1–14 years, with 4–5 years being more predominantly affected by PS (Caraballo et al., 2000). The most common symptoms exhibited by PS patients are full emetic triad (nausea, retching, vomiting), pallor, deviation in head and eyes, generalized seizures, ictal syncope (Yalçin and Toydemir, 2017), changes in thermoregulatory (Panayiotopoulos, 2005) and cardiorespiratory functions (Yamamoto et al., 2018). A very recent investigation on the incidence of PS recorded over 16 months in a population aged below 16 years old, reported 0.8/100,000 new cases. Similar study also recorded 6.1/100,000 new cases of BECTS within the same population, reporting 13 times higher prevalence of BECTS than PS (Weir et al., 2018).

The transient focal EEG abnormalities that are usually seen in the epilepsy, or also known as the “functional spikes” were initially thought to be confined to the occipital lobe in PS patients (Tsai et al., 2001). However, follow-up studies documented the functional spikes to shift to extra-occipital regions such as prefrontal and frontal (Kokkinos et al., 2010; Yoshinaga et al., 2010), parietal and lateral temporal lobe (Leal et al., 2008). This multifocal hyperexcitability nature of PS has led the researchers to correlate the affected cortical areas to the hypothalamic autonomic centers and the limbic system to the manifestation of transient hyperactive central autonomic network, which is the cardinal feature of PS (Ten Donkelaar and Horim, 2011). Generally, seizures in PS patients are sleep-related. Some studies have reported nearly all PS-associated seizures to take place during sleep (Caraballo et al., 2007), whereas some reported the 69.9% seizures to occur during sleep and 12.9% during awake (Specchio et al., 2010). More recent findings suggest the seziures are more likely to take place during awakenings (66.7%) (Yalçin and Toydemir, 2017). In sum, the constellation of findings suggests the PS-associated seizures to take place either during early awakening or sleeping hours.

The etiology of PS has been the subject of intense research, yet much of its pathophysiology has remained elusive. In 2007, a 12-year-old girl who presented with PS, was reported to carry a sporadic missense mutation in sodium voltage-gated channel alpha subunit 1 (*SCN1A)*, the gene encodes a voltage-gated sodium channel that was implicated in the pathology of Dravet Syndrome (Grosso et al., 2007). Two years later, another study reported two siblings with PS to have the *SCN1A* mutation. However, the father of the children also shared the *SCN1A* mutation, and surprisingly never experienced a seizure. This suggests that *SCN1A* mutation may merely increase the susceptibility to an idiopathic focal epilepsy phenotype (Livingston et al., 2009). In another study, 2 monozygotic twins presented with PS were found to have no mutations in the *SCN1A* gene or the *GABRG2* gene (another gene associated with Dravet Syndrome). Based on the early onset of PS in these patients and the severity of their symptoms, the authors concluded that mutations in the *SCN1A* gene may regulate the severity of the syndrome rather than the genesis of the disorder (Martín Del Valle et al., 2011). In a very recent study, a 6-year old girl diagnosed with PS was presented with a de novo 2.6 Mb deletion in 22q11.2 and an additional 172 kb duplication in 2q37.1 (Bertini et al., 2017). Deletion in 22q11.2 was associated with loss of genes involved in brain function and development, such as *RTN4R* (reticulon four receptor, NOGO RECEPTOR) (Pan et al., 2005; Ramasamy et al., 2014), *SNAP29* (synaptosomal-associated protein 29) and gene responsible for biogenesis of micro-mRNA, especially in mammalian brain such as *DGCR8* (microprocessor complex subunit 8) (Cheng et al., 2014). In addition to this, the 172 kb duplication in 2q37.1 was related to three genes, which include *ATG16L1* (Autophagy 16-like 1), *SAG* (S-antigen; retina and pineal gland), and *DGKD* (diacylglycerol kinase delta). Among these genes, *DGKD* coding for a cytoplasmic enzyme that phosphorylates diacylglycerol to produce phosphatidic acid has been implicated in epilepsy (Leach et al., 2007).

Similar to BECTS, children suffering from PS also demonstrate cognitive deficits, particularly in global visual-motor integration, writing, reading, arithmetic skills, verbal and visual-spatial memory (Germanò et al., 2005). The cognitive abnormalities reported in some of the PS patients was a result of the propagation of the interictal activity to various brain regions, including the frontal (Germanò et al., 2005) and parietal lobes (Lopes et al., 2014). In concordance with this finding, a more recent study reported changes in volume of prefrontal lobe and prefrontal-to-frontal lobe volume ratio in 3 PS patients who presented with status epilepticus. Conversely, the non- status epilepticus PS patients possess cortical growth pattern similar to that of healthy controls, suggesting the manifestation of SE in PS may impair the cognitive behavior of some PS patients (Kanemura et al., 2015).

## Pharmacotherapy for BECTS

The spontaneous remission of BECTS in adolescence has cast controversy over its treatment; in particular, as to whether or not to use anti-epileptic drugs (AEDs). A review of 110 recommendations from 96 published materials on BECTS revealed two-third of the findings to favor and one-thirds not to favor the use of AEDs. Most of those in favor with AEDs use, advocate for pharmacotherapy only in cases with early onset and multiple seizure expressions and also to limit the treatment to 1 year (Hughes, 2010). The need for AEDs was evident in some cases owing to the severity of the seizures, cognitive impairments and behavioral abnormalities that accompany the seizures in a large population of young children with BECTS (Kavros et al., 2008; Sarco et al., 2011; Samaitiene et al., 2012). In addition, there seem to be geographic differences in pharmacological management of BECTS. Sulthiamine (STM) was the most commonly prescribed AED in Austria and Germany (Gross-Selbeck, 1995). In the United States, the preferred AED for BECTS was carbamazepine (CBZ)/oxcarbazepine (OXZ) (Arzimanoglou and Wheless, 2007). European epileptologists prefer valproic acid (VPA) as the drug of choice for BECTS (Wheless et al., 2007).

There is a considerable amount of literature on VPA use in reducing electroclinical abnormalities (Gelisse et al., 1999; Xiao et al., 2014), increasing the threshold of motor evoked potentials (Nezu et al., 1997) and controlling epileptic negative myoclonus induced by other AEDs (Yang et al., 2008). In 2013, the ILAE recommended monotherapy with VPA for BECTS (level C evidence) (Glauser et al., 2013). Prolonged exposure to VPA was associated with weight gain in 40% of children (Corman et al., 1997). CBZ was shown to successfully treat BECTS populations in Japan (Oka et al., 2004), China (Ma and Chan, 2003), Greek (Gkampeta et al., 2015), and the United States (Wheless et al., 2005). Apart from reducing seizure incidences, CBZ also improved cognitive functions in BECTS patients by altering the epilepsy-induced changes in P300 event-related potential (Naganuma et al., 1994). One of the most undesirable side effects of CBZ was the drug-induced non-epileptic myoclonus and tic-like movements (Magaudda and Di Rosa, 2012). OXZ, a chemical twin of CBZ, was reported to normalize EEG, improved cognition and effectively controlled seizure expressions (Tzitiridou et al., 2005). It has a mild adverse effect profile such as headache and sedation (Coppola et al., 2007).

STM, a carbonic anhydrase inhibitor reduced spike and seizure frequencies (Wirrell et al., 2008), normalized EEG (Bast et al., 2003) with low seizure remission rate (91% success for 24 weeks of observation) (Borggraefe et al., 2013). Adverse effects such as impaired cognitive functions (Wirrell et al., 2008) and metabolic acidosis (Borggraefe et al., 2013) were associated with STM. On the other hand, LEV was shown to control seizures without (Bello-Espinosa and Roberts, 2003) and with minimum side effects (Verrotti et al., 2007). Few researchers reported that LEV improves BECTS-related impairments in auditory verbal memory and baseline auditory comprehension (Kossoff et al., 2007). The success rate of LEV on seizure remission was 81% as reported by Borggraefe et al. (2013). Adverse effects such as suicidal ideations (Borggraefe et al., 2013) and psychosis (Kossoff et al., 2001) were related to LEV use. Topiramate, a novel AED was shown to reduce epileptiform frequency and inhibit epileptiform discharges in BECTS patients. Its side effect includes anorexia, nausea, headache, and hypohidrosis / adiaphoresis (Liu et al., 2016).

## Pharmacotherapy for sleep related hypermotor epilepsy

CBZ has been documented as the most commonly prescribed drug to manage SHE (Provini et al., 1999; Gambardella et al., 2000). Almost two-thirds of the SHE patients responded well to bedtime low doses (200–1,000 mg/kg) of CBZ. However, one third of the patients remained resistant to the drug (Provini et al., 2000). High blood brain barrier penetrance of CBZ (Shorvon, 2000) indicates the propensity of the AED to react with a variety of neuronal receptors. In agreement with this, CBZ was shown to reduce the action potential frequency of voltage-gated sodium channels (McLean and MacDonald, 1986; Schwarz and Grigat, 1989) which in turn could alter the neuronal excitability by impairing the glutamate release (Sitges et al., 2007) or potentiating (gamma-Aminobutyric acid) GABA_A_ receptors (Zheng et al., 2009). More importantly, CBZ inhibits α4β2 and α2β4 subunits of nicotinic receptors (Di Resta et al., 2010) which have often been implicated in SHE. Therefore, it seems that inhibition of the nicotinic receptors by CBZ may suppress glutamate excitability and potentiate GABA activity in the thalamocortical system and hippocampus to attenuate hyperexcitability (Albuquerque et al., 2009; Aracri et al., 2010).

Many epileptologists reported better outcomes with OXZ in controlling nocturnal seizures (Raju et al., 2007; Romigi et al., 2008), even in patients unresponsive to CBZ and other AEDs (Raju et al., 2007). Similar to CBZ, OXZ block voltage-gated sodium channels (MacDonald and Rogawski, 2008), potentiate GABA_A_ receptors (Zheng et al., 2009) and inhibits α2β4 subunits of nicotinic receptors (Di Resta et al., 2010). In addition to OXZ, Oldani and co-researchers found administration of topiramate (50–300 mg daily at bedtime) as add-on (3 patients) or monotheraphy (21 patients) to reduce nocturnal seizures in 62.5% of patients and six of the total patients were seizure free in the follow-up that ranged from 6 months to 6 years. The authors reported mild adverse events include weight loss (6 patients), paresthesias (3 patients), and speech dysfunction (2 patients) which disappeared within 3 months (Oldani et al., 2006). More recently, in a Taiwanese series of 10 case studies, CBZ, OXZ, and topiramate along with other AEDs effectively reduced nocturnal seizures by 75% and abolished diurnal attack by more than 90% without producing any adverse effects (Yeh and Schenck, 2017).

Claudio Liguori and colleagues administered lacosamide as add-on therapy in 2 SHE patients that were unresponsive to other AEDs. Addition of lacosomide (200 mg/day) to polytherapy (CBZ+TPM) and (OXZ+clonazepam) dramatically abolished the nocturnal seizure expressions and both patients were then continued on lacosomide monotherapy for 12 months and remained seizures free (Liguori et al., 2016). In a separate study, administration of LCM (300–600 mg/kg) to eight patients with refractory-SHE reduced the seizure frequency in 5 patients for more than 50 and 25% in one patient. The authors also reported mild and reversible adverse events in most of the patients, such as transient fatigue and diplopia. However, one patient was withdrawn from lacosomide for feeling continuously “spaced out” (Samarasekera et al., 2018).

## Pharmacotherapy for panayiotopoulos syndrome

Most clinicians believe children with PS may not require prophylaxis therapy with AED (Panayiotopoulos, 2002, 2004). Nevertheless, 10–20% of PS patients face persistent autonomic status epilepticus that could last for days (García and Rubio, 2009) which place them at great risk of developing life threatening severe cardiorespiratory dysfunctions (Camfield and Camfield, 2005; Verrotti et al., 2005). In addition, there is a consensus among the epileptologists that AED therapy should be reserved for the patients with unusually frequent and severe form of seizures that could affect the quality of their lives (Ferrie et al., 2006). To date, there is no single monotherapy of any AED has been shown to be superior to the rest.

Garcia and Rubio have reported recurrence of seizures in some PS patients after 6 months of treatment with VPA. The same authors also showed LEV (1,000–2,000 mg/kg) that initially introduced as add-on therapy to these patients, and then as monotherapy, successfully reduced the occurrences of seizures and all the patients remained seizure free for 2–3 years (García and Rubio, 2009). In parallel to this finding, numerous studies have documented the potential advantages of LEV in pediatric epileptic cases owing to the AED's lack of interaction with other drugs, favorable elimination kinetics and significant protein binding ability (Leppik, 2001). In addition, LEV also lacks adverse effects such as weight gain, polycystic ovarian syndrome, hair loss, and rash that have been most frequently implicated in the use of VPA, CBZ and lamotrigine (Konishi et al., 1993; Barron et al., 2000). As mentioned earlier, long term use of LEV was associated with behavior-related adverse events (Kossoff et al., 2001; Borggraefe et al., 2013).

Rectal, buccal or intravenous (IV) benzodiazepines were commonly used to manage autonomic status epilepticus manifestations in PS (Ferrie et al., 2006). However, Lacroix et al. (2011) urged the clinicians to practice great caution over the use of BDZ to treat autonomic seizures. The authors reported severe respiratory depression following the benzodiazepines administration (diazepam 0.5–0.6 mg/kg, IR; lorazepam 0.05–0.06 mg/kg, IV) for seizures with autonomic manifestations in five patients. The authors went on to suggest that the use of other BDZ such as buccal midazalom, or a more autonomic tolerant AEDs such as VPA or LE for the acute management of autonomic seizures (Lacroix et al., 2011) should be considered.

## Future recommendations

The past decade has witnessed the birth of various AEDs. Despite their efficacy, they are not without severe adverse effects, especially in prolonged exposure to refractory epileptic patients. This calls for discovery of novel, more specific molecular-targeting pharmacotherapies. Therapeutic diets such as ketogenic diet and low-glycemic index diet were shown to be effective in treating drug-resistant epileptic patients (Pfeifer and Thiele, 2005; Neal et al., 2008). As well, adjunctive therapy with fenofibrate was shown to markedly reduce the seizure frequency in human and animal models of SHE (Puligheddu et al., 2017). Fenofibrates are agonists of peroxisome proliferator-activated receptor alpha (PPARα), which inhibits β2-containing nicotinic receptors by phosphorylating β2 (Melis et al., 2010; Puligheddu et al., 2013). In animal models of SHE, chronic diet with fenofibrates reduced the nicotine-induced spontaneous inhibitory postsynaptic current in pyramidal neurons of the frontal lobe. This subsequently attenuated the cholinergic overactivation and expressions of seizures (Puligheddu et al., 2017). Taken together, these findings warrant further investigation of the role of fenofibrate and PPARα in the pathogenesis of sleep-related epilepsy.

Identification of *GRIN2A* mutations in BECTS and other childhood epilepsies has thrown light on the role of GluN2A subunit-containing NMDARs in epilepsy (Lemke et al., 2013; Gao et al., 2017; Von Stülpnagel et al., 2017). Recent findings indicate that *GRIN2A* mutations prolong NMDARs' deactivation time, decrease the amplitude of current responses, reduce glutamate potency, reduce channel open probability and accentuate the sensitivity of NMDARs toward negative allosteric modulators (Gao et al., 2017; Sibarov et al., 2017). It is postulated that reduced NMDARs function may impair the inhibitory effects of GABAergic interneurons in the prefrontal cortex (Xi et al., 2009) and cerebral cortex (Bagasrawala et al., 2016); leading to epilepsy (Gao et al., 2017). In addition, NMDARs are also modulated by metabotropic glutamate receptor subtype 5 (mGlu5) (Chen H. H. et al., 2011). Negative modulation of mGlu5 has been promising in attenuating hyperexcitability of central nervous system (Kumar et al., 2013, 2016, 2017) and even reducing the spike-wave discharges in numerous animal models of epilepsy (McCool et al., 1998; Chapman et al., 2000). More studies are needed to further elucidate the functional link between mGlu5, GABA, and NMDARs in epilepsy. Discovery of mutations in *DEPDC5, NPRL2*, and *NPRL3* that encode for GATOR1 (negative modulator of mTORC1) have pioneered the channelopathy-independent approach in understanding the pathological process of NFLE. To date, mTORC1 inhibitors have only been proven successful in treating epilepsy in tuberous sclerosis (Curatolo, 2015) and polyhydramnios megalencephaly symptomatic epilepsy (Parker et al., 2013). Nevertheless, the potential functional link between mutations of mTORC1-related genes and non-lesional focal epilepsy (Myers and Scheffer, 2017) merits future studies.

We summarized the pharmacotherapy of options for BECTS, PS and SHE in Tables 2–4, respectively. The mechanism of actions for the AEDs is listed in Table 5.

**Table 2:** Table showing pharmacotherapy options for BECTS.

**Authors**	**Study^a^**	**Dose**	**Effects**	**Adverse effects**	**Adjunctive/Monotherapy**
**VALPROIC ACID**					
Gelisse et al., 1999	Case study	750 mg/day for 15 months	Reduce electrochemical abnormalities and seizures	None reported	Monotherapy
Xiao et al., 2014	Retrospective, uncontrolled, case-comparison cohort study	(9.3–27 mg/kg/day) followed at 6, 12, and 18 months	57.6% were seizure free at 6 months, 73.9% at 12 months, 100% at 18 months; 73.8% showed EEG normalization at 12 months, 95.7% at 18 months	Mild drowsiness (17.4%), mild weight gain (4.3%)	Monotherapy
**CARBAMAZEPINE**					
Ma and Chan, 2003	Retrospective observational study	(5–22.5) mg/kg for 3.35 years (mean)	Reduced seizure frequency	None Specific to CBZ was reported	Monotherapy
Kang et al., 2007	Multicenter, randomized, open-label, observer-blinded, parallel-group clinical trial	Started at 10 mg/kg/day and titrated to 20 mg/kg/day for over 22 weeks	Reduced seizure in 70% of patients, improved cognitive functions	Rashes, Weight gain (8.6% from initial weight)	Monotherapy
**TOPIRAMATE**					
Kang et al., 2007	Multicenter, randomized, open-label, observer-blinded, parallel-group clinical trial	Started at 12.5 mg per day and titrated to at least 50 mg per day in patients < 30 kg and 75 mg or 100 mg per day in patients >30 kg over 4 weeks.	Reduced seizure in 69.6% of patients	Memory dysfunction and somnolence	Monotherapy
Liu et al., 2016	Randomized control trial	Group A: started with 0.1–1 mg/kg/day to 2 mg/kg/day Group B: TPM given twice a day with final dose 4 mg/kg/day	Group A: overall seizure reduction efficacy was at 90.9% Group B: overall seizure reduction efficacy was at 92.5%	Group A: 8 (anorexia and nausea), 6 (headache and dizziness), 2 (weight loss), 2 (hypohidrosis / adiaphoresis) 1 (difficulty in finding words), 1 (long term fever and enuresis) Group B: 4 (light anorexia and nausea), 2 (dizziness), 1 (weight loss)	Monotherapy
**OXCARBAZEPINE**					
Tzitiridou et al., 2005	Open label, long term study	10 mg/kg/day (first week) increased to 20–25 mg/kg/day (second and third week), dosage was increased to 30 mg/kg/day (if the patients were unresponsive)	64% were seizure free; 21% experienced >50% improvement; 5% no improvement	None reported	Monotherapy
Coppola et al., 2007	Prospective, open label, pilot trial	5 mg/kg, followed by a 3-day titration at increments of 5 mg/kg, up to a maximum daily dose of 20 mg/kg	72.2% were seizure free	1 (headache), 1 (sedation) had to be withdrawn due to excessive sedation	Monotherapy
**LEVETIRACETAM**					
Coppola et al., 2007	Prospective, open label, pilot trial	5 mg/kg, followed by a 3-day titration at increments of 5 mg/kg, up to a maximum daily dose of 20 mg/kg	90.5% were seizure free	2 (decreased appetite), 1 (decreased appetite combined with daily frontal cephalalgia)	Monotherapy
Verrotti et al., 2007	Prospective, multicenter trial	Started at 250 mg/daily and titrated to 1,000–2,000 mg/daily. Followed for 12 months	42.8% patients were seizure free (started with levetiracetam); 30.1% patients were seizure free (patients unresponsive to other drugs, then followed up with levetiracetam)	Drowsiness and irritability in 9.5% of the patients	Monotherapy
Borggraefe et al., 2013	Randomized, double-blinded, controlled trial	Started at 10 mg/kg bodyweight and was further increased weekly by increments of 10 mg/kg weekly to a final dosage of 30 mg/kg bodyweight	81% of the patients were seizure free for 6 months; seizure recurrence in 19% patients	23.8% of the patients were dropped out due to suicidal ideation, headache, sleep disturbance, nausea, abdominal pain	Monotherapy
**SULTHIAME**					
Borggraefe et al., 2013	Randomized, double-blinded, controlled trial	Started at 2 mg/kg bodyweight and was further increased weekly by increments of 2 mg/kg bodyweight weekly to a final dosage of 6 mg/kg bodyweight	91% of the patients were seizure free for 6 months; seizure recurrence in 9.1% patients	4.1% (1) of the patients were dropped out due to adverse events related to airways.	Monotherapy

*CBZ carbamazepine*.

a *Study type or design*.

**Table 3:** Table showing pharmacotherapy options for Panayiotopoulos Syndrome.

**Authors**	**Study**	**Dose**	**Effects**	**Adverse** **effects**	**Adjunctive/Monotherapy**
**LEVETIRACETAM**					
García and Rubio, 2009	Case studies (3)	(1) Started with LEV (250 mg twice a day), increased to 500 mg every 12 h after 2 weeks, dose was increased to 1,000 mg (3 months after an attack) (2) Started with 250 mg twice a day of LEV and increased up to 1,000 mg for 2 weeks. (3) Started with LEV 250 mg, and increased up to 1,000 mg in 2 weeks. After two brief episodes, LEV 1,000 mg was continued twice/day for a year.	(1 and 2) The patient has been seizure free for 3 years. (3) The patient has not experienced any seizure from 13 to 16 years old.	None was reported	LEV was given as adjunctive with VPA. VPA was discontinued after 6 months' treatment with LEV [case 1 and 2] Monotherapy [case 3]
**VALPROIC ACID**					
Martín Del Valle et al., 2011	Case study	Treatment was started with valproic acid and followed for 2 years (dose not mentioned in the study)	The patient was seizure free for 2 years; Partial improvement in the patient's EEG was reported	None was reported	Monotherapy

*LEV, levetiracetam; EEG, electroencephalogram*.

*^a^Study type or design*.

**Table 4:** Table showing pharmacotherapy options for sleep related hypermotor epilepsy.

**Authors**	**Study^a^**	**Dose**	**Effects**	**Adverse effects**	**Adjunctive/Monotherapy**
**POLYTHERAPY**					
Yeh and Schenck, 2017	Case studies (10)	2 cases: Carbamazepine from 200 to 800 mg/day in monotherapy; 3 cases: polytherapy (combination of topiramate or lamotrigine). 4 cases: Oxcarbamazepine from 300 to 1,200 mg/day in polytherapy (2 with topiramate, 1 with topiramate and acetazolamide) One case: acetazolamide and clonazepam; One case: sodium valproate 300 mg bid and levetiracetam, 500 mg	75% reduction in nocturnal seizures and abolishment of occasional diurnal attack by more than 90%	None was reported	Adjunctive
**LACOSAMIDE**					
Samarasekera et al., 2018	Case study	Lacosamide was initiated at dosage ranging from 300 to 600 mg/day for 6–37 months.	Five patients showed more than 50% reduction in seizure expressions; One patient showed 25% response; Two patients withdrawn from lacosamide after 2 and 24 months	2 patients: Transient fatigue within the first 6 months 1 patient: was feeling “spaced out” 1 patient: experienced diplopia at 500 mg/day and symptoms resolved after the dosage reduced	Adjunctive lacosamide was given in adjunct to 6 other drugs such as carbamazepine, topiramate, Oxcarbazepine, Phenytoin, valproate and zonisamide
Liguori et al., 2016	Case study (2)	Lacosamide 200 mg/day was given along carbazepine, topiramate (case 1) and oxcarbazepine, clonazepam (case 2); After seizure reduction, lacosamide was maintained on monotherapy and followed up for 1 year	Both patients were seizure free at 12 months' follow up	None was reported	Adjunctive/Monotherapy
**OXCARBAZEPINE**					
Raju et al., 2007	Case study (8)	Started oxcarbazepine at 10 mg/kg/day twice/daily and the dose was increased to 15–45 mg/kg/day	Six patients: seizure reduced within 4 days and under control in 2 weeks; Two patients: seizure under control at higher dose	Dizziness, somnolence, and diplopia in 2 patients	Adjunctive/ Monotherapy
Romigi et al., 2008	Case study	Oxcarbazepine was started at 10 mg/day/kg and increased to 20 mg/kg/day in 10 days and followed up for 4 months	Nocturnal seizures were completely disappeared	None was reported	Monotherapy
**TOPIRAMATE**					
Oldani et al., 2006		Topiramate was given as monotherapy in 21 patients: dosage ranging from 50 to 300 mg daily at bedtime and followed up from 6 months to 6 years. Topiramate was administered as add on to carmazepine in 3 patients.	6 patients were seizure free; 15 responders and 3 non-responders.	Weight loss (6), paresthesias (3), speech dysfunction in phonematic verbal fluency (2). All adverse events disappeared within 3 months	Adjunctive/ Monotherapy

a *Study type or design*.

**Table 5:** Table showing the mechanism of actions for anti-epileptic drugs.

**Anti-epileptic Drug**	**Mechanism of action(s)**
Sulthiamine	Acidification of brain tissue via inhibition of carbonic anhydrase (Tanimukai et al., 1964) Reduction in intracellular pH level, affects the opening of ion channels such as Na^+^/H^+^ exchange and Cl^−^/${\text{HCO}}_{3}^{-}$ exchanger (Bonnet and Wiemann, 1999; Bonnet et al., 2000)
Topiramate	Affects voltage gated sodium conductance (Zona et al., 1997) Block AMPA receptor activity (glutamate) (Coulter et al., 1993) Inhibits the release of glutamate (Hanaya et al., 1998) Anti-cholinergic effects (Avoli et al., 1998) Affects high-voltage N and L-type Ca^2+^ currents (Zhang et al., 1998)
Valproic acid	Stimulates glutamine synthetase (Phelan et al., 1985) Reduces the activity of phosphatidylinositol (3,4,5)-trisphosphate (Chang et al., 2014) Prevents hyperexcitability by acting on Kv7.2 channel and A-kinase anchor protein 5 (Kay et al., 2015) Blockade of NMDAR mediated current (Gean et al., 1994) Increases the brain level of GABA (Bertelsen et al., 2018)
Carbamazepine	Inhibits firing of cortical neurons by blocking the voltage gated sodium channel (Panayiotopoulos, 2005) Inhibits L-type calcium channels (Panayiotopoulos, 2005)
Oxcarbamazepine	Blocks voltage sensitive sodium channels (Panayiotopoulos, 2005)
Levetiracetam	Modulates synaptic vesicle glycoprotein 2A (Lynch et al., 2004) Inhibits presynaptic calcium channels (Vogl et al., 2012)
Lacosamide	Enhances slow sodium channel inactivation (Rogawski et al., 2015)

*AMPA, α-amino-3-hydroxy-5-methyl-4-isoxazolepropionic acid; NMDAR, N-methyl-D-aspartate; GABA, gamma-Aminobutyric acid*.

*Kv7.2 was referring to principal molecular components of the slow voltage-gated M-channel*.

## Author contributions

JK, AS, PM, and SD conceived the idea. JK, AS, PM, SD, and RM wrote the manuscript. JK and SD prepared the tables. JK, PM, and SD edited the manuscript.

### Conflict of interest statement

The authors declare that the research was conducted in the absence of any commercial or financial relationships that could be construed as a potential conflict of interest.

## Abbreviations

ILAE: International League Against Epilepsy
SHE: sleep related hypermotor epilepsy
BECTS: benign partial epilepsy with centrotemporal spikes
PS: Panayiotopoulos Syndrome
REM: rapid eye movement
NREM: non-rapid eye movement
Ca^2+^: calcium
K^+^: potassium
NFLE: Nocturnal Frontal Lobe Epilepsy
nAChRs: nicotinic acetylcholine receptors
CHRNA2: cholinergic receptor nicotinic alpha 2 subunit
CHRNA4: cholinergic receptor nicotinic alpha 4 subunit
CHRNB2: cholinergic receptor nicotinic beta 2 subunit
GABA: gamma-Aminobutyric acid
Ach: acetylcholine
PRIMA1: proline rich membrane anchor 1
Cl−: chloride
NKCC1: Na+/K+/Cl− co-transporter-1
KCC2: K+/Cl− co-transporter-2
PFC: prefrontal cortex
KCNT1: potassium-sodium activated channel subfamily T member 1
KCNT2: potassium-sodium activated channel subfamily T member 1
DEPDC5, Disheveled: Eg-10 and Pleckstrin Domain containing proteins
GATOR1: Gap Activity Toward Rags 1
mTOR: mammalian target of Rapamycin
mTORC1: mTOR Complex 1
NPRL2: nitrogen permease regulator-like 2
NPRL3: nitrogen permease regulator-like 3
RE: rolandic epilepsy
PRRT2: proline-rich transmembrane protein gene
ELP4: elongator acetyltransferase complex subunit 4
SRPX2: sushi-repeat containing protein X-linked 2
ARE: atypical RE
NMDARs: N-methyl-D-aspartate receptors
GRIN2A: α2 subunit of N-methyl-D-aspartate receptors
GABRG2: gamma-aminobutyric acid receptor subunit gamma-2
RBFOX1: RNA Binding Fox-1 Homolog 1
RBFOX3: RNA Binding Fox-1 Homolog 3
SCN1A: sodium voltage-gated channel alpha subunit 1
RTN4R, reticulon four receptor: NOGO RECEPTOR
SNAP29: synaptosomal-associated protein 29
DGCR8: microprocessor complex subunit 8
ATG16L1: Autophagy 16-like 1
SAG: S-antigen
retina and pineal gland
DGKD: diacylglycerol kinase delta
STM: sulthiamine
CBZ: carbamazepine
OXZ: oxcarbazepine
VPA: valproic acid
LEV: levetiracetam
PPARα: peroxisome proliferator-activated receptor alpha.
